# UDP-Glucose 4-Epimerase and β-1,4-Galactosyltransferase from the Oyster *Magallana gigas* as Valuable Biocatalysts for the Production of Galactosylated Products

**DOI:** 10.3390/ijms19061600

**Published:** 2018-05-29

**Authors:** Hui-Bo Song, Meng He, Zhi-Peng Cai, Kun Huang, Sabine L. Flitsch, Li Liu, Josef Voglmeir

**Affiliations:** 1 Glycomics and Glycan Bioengineering Research Center (GGBRC), College of Food Science and Technology, Nanjing Agricultural University, Nanjing 210095, China; 2013208001@njau.edu.cn (H.-B.S.); 2015108040@njau.edu.cn (M.H.); caizhipeng@njau.edu.cn (Z.-P.C.); 2 Department of Food Science, Zhejiang Pharmaceutical College, Ningbo 315100, China; 3 Manchester Institute of Biotechnology, University of Manchester, Manchester M1 7DN, UK; kun.huang-3@manchester.ac.uk (K.H.); sabine.flitsch@manchester.ac.uk (S.L.F.)

**Keywords:** UDP-galactose, UDP-glucose 4-epimerase, *Magallana gigas*, oyster metabolism

## Abstract

Uridine diphosphate galactose (UDP-galactose) is a valuable building block in the enzymatic synthesis of galactose-containing glycoconjugates. UDP-glucose 4-epimerase (UGE) is an enzyme which catalyzes the reversible conversion of abundantly available UDP-glucose to UDP-galactose. Herein, we described the cloning, expression, purification, and biochemical characterization of an unstudied UGE from the oyster *Magallana gigas* (MgUGE). Activity tests of recombinantly expressed MgUGE, using HPLC (high-performance liquid chromatography), mass spectrometry, and photometric assays, showed an optimal temperature of 16 °C, and reasonable thermal stability up to 37 °C. No metal ions were required for enzymatic activity. The simple nickel-affinity-purification procedure makes MgUGE a valuable biocatalyst for the synthesis of UDP-galactose from UDP-glucose. The biosynthetic potential of MgUGE was further exemplified in a coupled enzymatic reaction with an oyster-derived β-1,4-galactosyltransferase (MgGalT7), allowing the galactosylation of the model substrate *para*-nitrophenol xylose (*p*NP-xylose) using UDP-glucose as the starting material.

## 1. Introduction

**Scheme 1:**
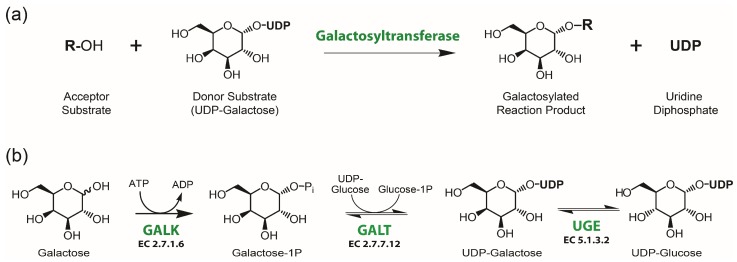
General schemes of an (**a**) enzymatic galactosylation reaction, and (**b**) the biosynthesis of uridine diphosphate glucose (UDP-glucose) from galactose via the actions of galactokinase (GALK), galactose 1-phosphate uridylyltransferase (GALT), and UDP-glucose 4-epimerase (UGE).

Galactose-containing carbohydrates are widely distributed in living systems, and play important biological roles as structural elements, energy sources, and precursors for cell surface elements. In most organisms, the decoration of specific acceptor molecules with galactose is catalyzed by galactosyltransferases [1,2]. These enzymes transfer galactose from the activated donor substrate UDP-galactose [3] to acceptors such as glycoproteins [4], glycolipids [5], lipopolysaccharides [6], or polysaccharides [7] (Scheme 1a). Therefore, galactose activation, through which galactose is converted into its active form uridine diphosphate galactose (UDP-galactose), is essential prior to the formation of galactose-modified conjugates. This procedure is catalyzed by the enzyme UDP-glucose 4-epimerase (UGE). Thus, UGE is an enzyme critical for numerous biological processes.

In addition to the biosynthetic role of UGE in carbohydrate synthesis, this enzyme is also a pivotal part of the Leloir pathway, which metabolizes galactose through glycolysis [8]. This interconversion of galactose into glucose 1-phosphate is accomplished by the consecutive actions of galactokinase (yielding galactose 1-phosphate) [9], galactose 1-phosphate uridylyltransferase (catalyzing the exchange of the phosphate group with the UDP group from UDP-glucose, yielding UDP-galactose and glucose 1-phosphate) [10], and UGE (interconverting UDP-galactose back into UDP-glucose) [11], resulting in the full catabolic removal of galactose in vivo (Scheme 1b). In humans, functional deficiencies in the Leloir pathway result in a severe metabolic disorder known as galactosemia (the molecular aspects of galactosemia [12,13] were recently reviewed by Timson [14]).

The mimicking of physiologically relevant processes via in vitro studies is an approach generally employed to better understand the role of galactosylation in living organisms and requires access to the relevant enzymes, including UGE. Also, various galactose-containing glycoconjugates, derived from marine mussels and other molluscs, were found to bear critical biological functions when used as supplements [15]. UGE from the oyster *Magallana gigas* (MgUGE) is required for the biosynthesis of the sole precursor (UDP-galactose) for the biosynthesis of all galactose-containing glycoconjugates in the Pacific oyster, and thus, was an interesting target for further elucidation.

Several UGE isoforms originating from various plants, animals, yeasts, and bacteria were reported throughout the last 25 years. However, the biotechnological potential of carbohydrate-modifying enzymes from molluscs in general, and oysters in particular is yet to be explored. Recent progress in the genetic analysis of the Pacific oyster inspired us to mine the now publicly available genome dataset of *Magallana gigas* [16] for industrially relevant glycoenzymes with potentially interesting enzymatic features. The work presented herein is the first description and characterization of a carbohydrate oxidoreductase derived from molluscs.

## 2. Results

### 2.1. Cloning and Homology Analysis of the Oyster UGE Gene

**Figure 1:**
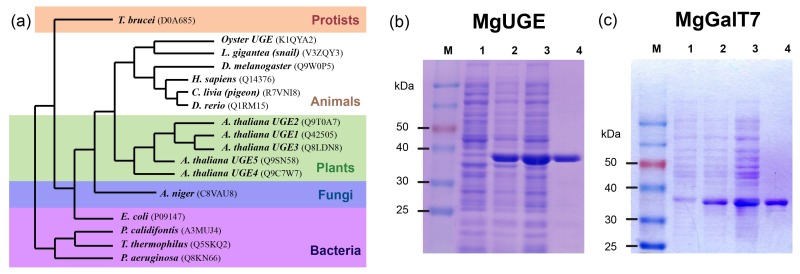
(**a**) Phylogenetic relationship of functionally characterized UGEs, with UniProt identifiers shown in parentheses. SDS-PAGE analyses of recombinant (**b**) UGE from *Magallana gigas* (MgUGE), and (**c**) β-1,4-galactosyltransferase from *Magallana gigas* (MgGalT7), at various stages of expression and purification. **M**—protein marker; **1**—cell pellet before induction, **2**—cell pellet after induction with isopropyl-β-d-thiogalactopyranoside (IPTG), **3**—supernatant after cell lysis, and **4**—purified protein.

A gene encoding a putative UGE candidate was selected by doing a basic local alignment search (BLAST) of the Pacific oyster transcriptome database against the human UGE sequence (UniProt ID: Q14376). The full-length open reading frame (ORF) of the encoding gene was successfully cloned, and consisted of 1023 base pairs (Figure S1). A homology search revealed that MgUGE is closely related to UGE isoforms from other animals, particularly to UGE from the sea snail *Lottia gigantea* (UniProt ID: Q9W0P5), and to lesser extents, the human, fruit fly, pigeon, and zebrafish UGE isoforms (UniProt IDs: Q14376, Q9W0P5, R7VNI8, and Q1RM15, respectively). As one can expect, UGE isoforms of plant, protist, fungal, or bacterial origins showed fewer similarities to MgUGE (Figure 1a).

### 2.2. Protein Expression and Purification

The recombinant gene product of MgUGE was successfully expressed in a soluble form, as judged by SDS-PAGE (Figure 1b). The fused hexahistidine tag further allowed the recombinant MgUGE protein to be purified to near homogeneity. The purified sample migrated as a single protein band with a molecular weight between 35 and 40 kDa, which was in good agreement with the calculated mass of 38.6 kDa (including the 1.3 kDa from the added C-terminal hexahistidine tag). The protein concentration of the purified MgUGE was determined to be 3.45 ± 0.05 mg/mL, and the specific activity was calculated to be 3.48 U/mg.

β-1,4-Galactosyltransferase from *Magallana gigas* (MgGalT7, UniProt ID: K1QT40) was also successfully expressed and purified in a soluble form (Figure 1c). Using SDS-PAGE, a predominant protein band was observed at a size which was in good agreement with the theoretical mass of 37.8 kDa (including the 5.4 kDa from the N-terminal hexahistidine tag).

### 2.3. Biochemical Characterization

MgUGE activity was tested in a coupled enzymatic assay (Figure S2); MgUGE interconverts the substrate UDP-galactose into UDP-glucose in a first step, where UDP-glucose can be further oxidized in the presence of NAD^+^ by UDP-glucose dehydrogenase (StUGD) to UDP-glucuronic acid and NADH (nicotinamide adenine dinucleotide; note: UDP-galactose cannot be oxidized by StUGD). The strong absorbance of NADH at 340 nm is, therefore, suitable to follow the coupled enzymatic reaction in a photometric assay using a plate reader.

**Figure 2:**
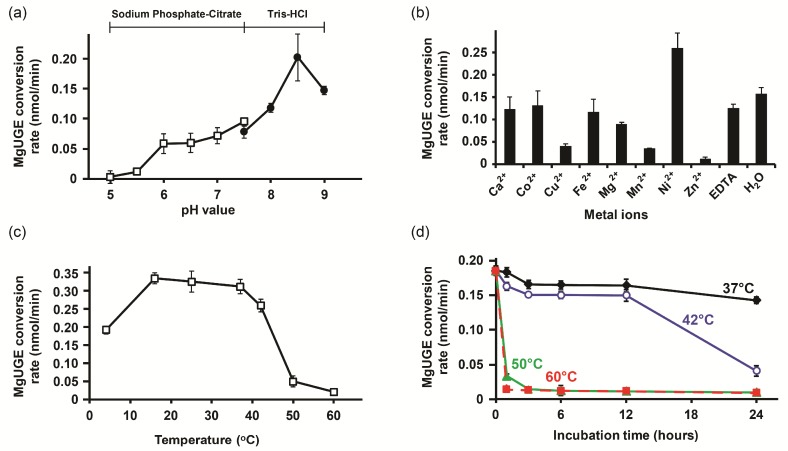
Biochemical characterization of MgUGE. (**a**) pH dependency of recombinant MgUGE; (**b**) Impact of metal ions and ethylenediaminetetraacetic acid (EDTA) on the enzymatic activity of MgUGE; (**c**) MgUGE activity at various incubation temperatures; and (**d**) Thermal stability of MgUGE using multiple pre-incubation times and temperatures. The pH values of the Tris-HCl and sodium phosphate-citrate buffers represent their pH values at 22 °C. The error bars represent the standard deviations calculated from three independent experiments.

MgUGE showed good activity in a pH range of 8.0 to 9.0, but showed a rapid decrease in activity outside of this range (Figure 2a). The addition of Ca^2+^, Co^2+^, Fe^2+^, or Mg^2+^ ions, or ethylenediaminetetraacetic acid (EDTA) did not affect the enzyme’s activity, while the addition of Cu^2+^, Mn^2+^, and Zn^2+^ ions partially inhibited the activity of MgUGE. Interestingly, the addition of Ni^2+^ ions slightly increased the activity of MgUGE (Figure 2b). The optimal reaction temperature of MgUGE was determined to be 16 °C (Figure 2c). Above 16 °C, over 80% of enzymatic activity was maintained, while increasing temperatures up to 37 °C. Reactions performed above 42 °C showed significantly reduced enzymatic activities. The enzyme showed reasonable thermal stability when incubated at 42 °C or below for up to 24 h, but showed almost complete inactivation within 1 h at 50 °C or above (Figure 2d). No significant effect on the activity of MgUGE was observed when adding various concentrations of 2-mercaptoethanol, urea, or imidazole to the reaction mixture (Figure S3). However, MgUGE was fully inactivated by the addition of low concentrations (0.1% *w*/*v*) of sodium dodecyl sulfate (SDS). The *K*_m_ value of MgUGE was 1.6 ± 0.1 mM for UDP-galactose, the ν_max_ value was 270 ± 9 µM∙min^−1^, and the *k*_cat_ value was calculated to be 134 ± 12 min^−1^ (Figure S4).

### 2.4. Enzymatic Galactosylation Reaction of Para-Nitrophenol Xylose

**Figure 3:**
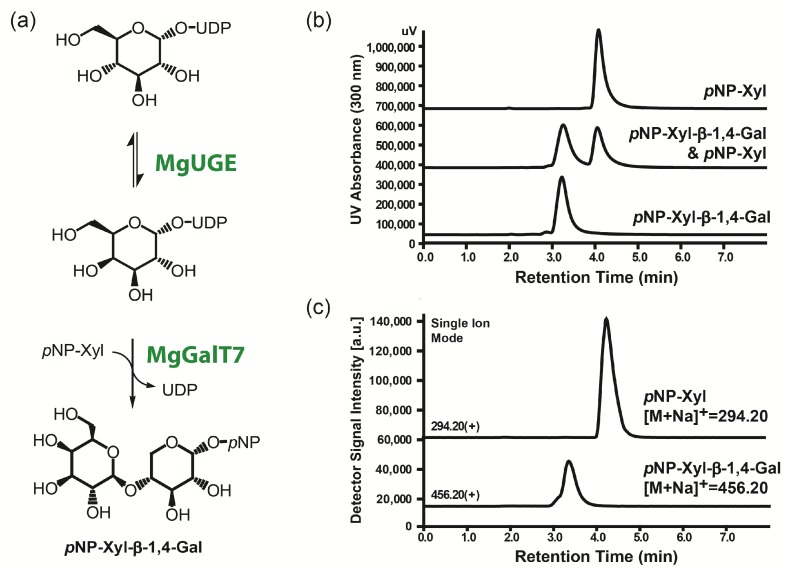
Enzymatic galactosylation reaction. (**a**) Schematic overview of the reaction cascade consisting of MgUGE and MgGalT7 using *para*-nitrophenol xylose (*p*NP-xylose) and UDP-Glucose as substrates; and (**b**) HPLC and (**c**) mass spectrometric analyses of the acceptor substrate and the galactosylated reaction product.

The biosynthetic potential of MgUGE was exemplified in an enzymatic galactoside synthesis reaction, by using MgUGE in conjunction with an oyster-originated β-1,4-galactosyltransferase (MgGalT7), allowing the galactosylation of the model substrate *para*-nitrophenol xylose (*p*NP-xylose). The success of this galactosylation reaction was examined through HPLC (high performance liquid chromatography) and HPLC/electrospray ionization MS (HPLC-ESI-MS) analysis. The UV-absorbance of these aromatically modified acceptor substrates (absorbance maximum of *p*NP-xylose is ~300 nm) facilitated the analyses of the analytes by HPLC-ESI-MS (Figure 3a). HPLC analysis of the incubated reaction mixture showed a second distinct HPLC peak (Figure 3b), which was collected, dried, and then subjected to mass spectrometric analysis. The mass value of the isolated HPLC peak was determined by ESI-MS in positive ionization mode, and clearly identified the expected product mass ([M + H]^+^ of *m*/*z* = 456.20), which was one galactose unit greater than the mass of the acceptor substrate *p*NP-xylose ([M + H]^+^ of *m*/*z* = 294.20, Figure 3c).

## 3. Discussion

### 3.1. Characterization of MgUGE

The recombinant form of MgUGE was successfully purified, and was observed as a single protein band of expected molecular weight with SDS-PAGE. Furthermore, analyses using a microplate reader, HPLC, and HPLC-ESI-MS unambiguously demonstrated the enzymatic epimerization activity of MgUGE. Good expression levels were achieved using *Escherichia coli* (*E. coli*) BL21-DE3 lacZ^−^ as an expression host. Unexpectedly, the initial comparative trials of expression and activity showed that both the expression level and activity of MgUGE were significantly higher than a recombinantly overexpressed UGE isoform from *E. coli* (UniProt ID: P09147).

The pH optimum of MgUGE lay in the range of most characterized epimerases, such as the isoforms from *Hordeum vulgare* (pH 8–9.5) [17], *Arabidopsis thaliana* (pH 8.6) [18], *Aspergillus nidulans* (pH 8.5) [19], and *E. coli* (pH 8.0) [20]. The UGE isoforms from the plant *Vicia faba* and from humans were reported to have a higher pH optimum of 9.5 [21,22]. The addition of Cu^2+^, Mn^2+^, and Zn^2+^ ions significantly reduced the enzymatic activity of MgUGE. Interestingly, similar effects were reported for the *Pisum sativum* UGE for Cu^2+^ and Zn^2+^ ions, whereas Mn^2+^-ions were reported to have had no negative impact on enzymatic activity [18]. EDTA did not reduce or inhibit MgUGE activity, which indicated that metal ions were not required in the catalytic mechanism of the enzyme. These findings were in agreement with previously reported biochemical and crystallographic data on UGEs [10,20]. The optimal temperature of MgUGE was determined to be 16 °C. However, good enzymatic activities were still measured at temperatures up to 37 °C, which correlated well with the temperature range of the Pacific oyster’s natural environment, ranging from 8 to 30 °C [23]. A similar temperature optimum of 15 °C was also reported for a UGE isoform from *Pisum sativum* [18], whereas other plant UGE isoforms from *Arabidopsis thaliana*, and the human UGE isoform showed temperature optima of 35 °C and 37 °C, respectively [22,24]. Although MgUGE showed no activity at temperatures of 50 °C and above, the enzyme showed reasonable stability when incubated at 37 °C for up to 24 h, which makes MgUGE an interesting candidate for biosynthetic applications at ambient temperatures with longer incubation times.

The kinetic analysis showed that the measured *K*_m_ value of 1.6 mM for UDP-galactose was higher when compared with other reported *K*_m_ values (between 0.0086 and 0.53 mM) [21,25,26,27], which might be an advantage for bioconversion reactions performed at higher substrate concentrations.

### 3.2. Potential of MgUGE in Galactosylation Reactions

The potential benefit of MgUGE in the synthesis of galactose-containing oligosaccharides was exemplified by the successful synthesis of *p*NP-xylose-galactose from the starting materials UDP-glucose and *p*NP-xylose. The reason why *p*NP-xylose was selected as the acceptor was due to the strict substrate specificity toward xylose of the employed β-1,4-galactosyltransferase (MgGalT7) which was also cloned for this work from oysters. The generation of the galactosylated product indicated good enzymatic activities of MgUGE and MgGalT7, and thus, showed the potential of these biocatalysts for coupled enzymatic galactosylation reactions. The combination of MgUGE with other galactosyltransferases derived from oysters or other species needs to be further evaluated so as to expand the scope of acceptor substrates.

### 3.3. Molecular Mechanism of MgUGE

The catalytic mechanism of human and bacterial UGE isoforms was extensively studied in the past [12,13,28]. It features the oxidation at the C4-position of UDP-glucose by NAD^+^, leading to UDP-4-keto-glucose and NADH, the rotation of the 4-keto-glucose moiety by 180° within the UGE binding site, and the reduction of the rotated 4-keto-glucose moiety, yielding UDP-galactose and NAD^+^ (Figure S5a) [27,29]. A schematic two-dimensional model of the UDP-glucose and NADH binding sites of MgUGE is illustrated in Figure S5b,c, showing a total of 15 hydrogen bond interactions, and 26 hydrophobic interactions. The tyrosine and lysine residues, which are part of a conserved Y-X-X-X-K motif, were found essential for the catalytic activity of human and fungal UGE isoforms [19,28], and are also present in MgUGE (Figure S6). Mutational studies of these residues in the *A. nidulans* mutant isoforms, replacing tyrosine at position 156 (Tyr156) with phenylalanine, and lysine at position 160 (Lys160) with valine, completely abolished the activity of the enzyme. Given the close homology of all UGE isoforms, we presume that the corresponding Tyr152 and Lys162 residues of MgUGE are also essential for the interconversion of UDP-glucose to UDP-galactose.

## 4. Materials and Methods

### 4.1. General

Restriction endonucleases, and T4 ligase were purchased from Thermo Fisher Scientific (Shanghai, China). Primestar DNA polymerases were purchased from Takara (Dalian, China). DNA Gel Purification and Plasmid Extraction kits were purchased from Axygen (Beijing, China). *Escherichia coli* Mach1 T1 cells (Life Technologies, Shanghai, China) were used for plasmid amplification and manipulation procedures. A β-galactosidase-deficient *E. coli* BL21-DE3 knockout strain (lacZ^−^) was generated using the λ-Red recombinase system, as described by Datsenko and Wanner [30]. DNA primers were obtained from GenScript (Nanjing, China). TRIzol was purchased from Invitrogen (Thermo Fisher Scientific, Shanghai, China). Buffer salts, and other chemicals of the highest available grade were purchased from various local suppliers.

### 4.2. Gene Amplification and Construction of the Expression Vectors

Fresh Pacific oyster samples were used to prepare complementary DNA (cDNA). Briefly, 75 mg of homogenized oyster tissue was mixed with 1 mL of TRIzol reagent. The mixture was centrifuged at 14,000× *g* for 15 min, and the clear supernatant was mixed with 200 µL of chloroform. After another centrifugation step at 14,000× *g* for 15 min, the upper aqueous layer was mixed with 500 µL of isopropanol, before being left on ice for 20 min, and then again centrifuged at 14,000× *g* for 8 min. The resulting RNA pellet was washed in 1 mL of 75% ethanol (EtOH), and after drying, was dissolved in 25 µL of water treated with diethylpyrocarbonate (DEPC). The isolated oyster RNA was then used for the generation of cDNA, using the Promega Reverse Transcription System (Promega Biotech, Beijing, China), according to the manufacturer’s instructions.

The oligonucleotide primers for amplifying the putative *MgUGE* and *MgGalT7* genes were based on the sequencing information of the Pacific oyster transcriptome [16], and were designed as follows: 5′-TCATATGGGAGACAGCTGCATCTTGG-3′ (*MgUGE* forward primer), 5′-TCTCGAGTTTTCGGAAACCGTTTGGATTC-3′ (*MgUGE* reverse primer), 5′-CGGATCCAAGCTACAGAGTGATAC-3′ (*MgGalT7* forward primer), and 5′-ACTCGAGTTATGGTATTAGTCCTTTAG-3′ (*MgGalT7* reverse primer). The gene amplifications were carried out using Primestar HS DNA polymerase (Takara, Dalian, China), based on the manufacturer’s instructions. Briefly, the PCR amplification was performed using 30 PCR cycles (for *MgUGE*), and 28 PCR cycles (for *MgGalT7*), consisting of denaturation at 95 °C for 10 s, annealing at 58 °C (for *MgUGE*) and 56 °C (for *MgGalT7*) for 30 s, and elongation at 72 °C for 45 s. The PCR fragments were purified using agarose gel electrophoresis (Figures S1 and S7), then digested with the restriction endonucleases Nde *I* and Xho *I* (for *MgUGE*), or Bam *I* and Xho *I* (for *MgGalT7*), and subsequently ligated into predigested pET-30a expression vectors. The ligation mixtures were transformed into *E. coli* Mach1 T1 competent cells, and were placed on Luria–Bertani (LB) agar plates containing 50 µg/mL kanamycin, for the selection of positive transformants. Colonies were screened by Sanger DNA sequencing (Genscript, Nanjing, China), and clones containing the expected plasmid construct were stored at −80 °C, and used for further experiments. Extraction of plasmids, endonuclease treatments, ligation, DNA purification, and transformation were carried out using standard protocols.

### 4.3. Expression and Purification of Recombinant MgUGE and MgGalT7

DNA constructs were transformed by electroporation into the *E. coli* BL21-DE3 lacZ^−^ expression host, and a single grown colony was then inoculated in 400 mL of sterile LB medium at 37 °C, and shaken at 250 rpm until the culture reached an absorption value at 600 nm (OD_600_) of 0.5–0.6. The incubation temperature was then reduced to 25 °C, and isopropyl-β-d-thiogalactopyranoside (IPTG) was added to attain a final concentration of 1 mM. After 6 h of induction, the cells were harvested by centrifugation (5000× *g* for 15 min). After re-suspending the cell pellet in 10 mL lysis buffer (100 mM NaCl, 50 mM Tris, 1% Triton X-100, 1 mM phenylmethylsulfonyl fluoride (PMSF), adjusted with HCl to pH 8.0), cells were disrupted by sonication for 20 min (40 on/off cycles with 20 µm amplitude for 15 s). The resulting cell lysate was cleared by centrifugation (20,000× *g* for 20 min). The supernatant was loaded onto a Ni-NTA affinity column (Ni^2+^-nitrilotriacetate, 2 mL bed volume), then equilibrated with binding buffer (50 mM NaCl, 50 mM Tris/HCl, pH 8.0), and washed with 10 bed volumes of washing buffer (50 mM NaCl, 10 mM imidazole, 50 mM Tris/HCl, pH 8.0) to remove unspecifically bound remnants. The histidine-tagged MgUGE or MgGalT7 proteins were eluted using an elution buffer (50 mM NaCl, 250 mM imidazole, 50 mM Tris/HCl, pH 8.0), and collected in 1 mL fractions. Relevant fractions were analyzed by SDS-PAGE under denaturation conditions, and visualized using Coomassie Brilliant Blue G-250 staining (Alfa Aesar, Shanghai, China). Elution fractions of MgUGE which showed the highest homogeneity on a protein gel were pooled, and quantified using the Bradford protein quantification method [30].

### 4.4. Enzymatic Assay

The activity of MgUGE was determined using a photometric assay, based on the method described by Franke et al. [31] with slight modifications. The activity test relied on the activity of MgUGE to convert UDP-galactose to UDP-glucose, which was subsequently oxidized in the presence of NAD^+^ by *Sphaerobacter thermophilus* UDP-glucose dehydrogenase (StUGD), forming UDP-glucuronic acid and NADH [32] (Figure 3). Typically, the reaction mixtures (30 μL) consisted of 14.4 μg of purified MgUGE, UDP-galactose (2 mM), MgCl_2_ (2 mM), and 50 mM Tris/HCl buffer (pH 8.5). Reaction mixtures lacking either MgUGE or UDP-galactose were used as negative controls. After 10 min of incubation at 37 °C, the reaction was quenched by heat inactivation (10 min, 95 °C), before 20 μL of an oxidizing solution containing 15 mM NAD^+^, 4 μg of StUGD, and 100 mM Tris/HCl (pH 8.5) was added. Absorbance changes at 340 nm due to the formation of NADH were continuously recorded over a period of 60 min using a microplate reader (Thermo Multiskan, Shanghai, China) at 37 °C. Standard deviations were calculated from three independently performed experiments. One unit of MgUGE activity was defined as the amount of enzyme which converts 1 μmol of UDP-galactose to UDP-glucose within 1 min at 37 °C.

### 4.5. Biochemical Characterization of MgUGE

The biochemical parameters of MgUGE were determined using the plate-reader-based assay described in Section 4.4. The conversion of UDP-galactose to UDP-glucose did not exceed 20% in any of the reactions. The pH optimum of MgUGE was determined using sodium phosphate-citrate buffer (final concentration 50 mM, between pH 5.0 and pH 7.5), and Tris-HCl buffer (final concentration 50 mM, between pH 7.5 and pH 9.0) at 22 °C. Optimal reaction temperatures were determined in 50 mM Tris-HCl buffer (pH 8.0) in a temperature range of 4 °C to 60 °C. The thermal stability assays were carried out by pre-incubating MgUGE in 50 mM Tris-HCl buffer (pH 8.0) at 37 °C, 42 °C, 50 °C, and 60 °C for various time periods up to 24 h. The effects of various metal ions (Ca^2+^, Co^2+^, Cu^2+^, Fe^2+^, Mg^2+^, Mn^2+^, Ni^2+^ and Zn^2+^, all added in their chloride forms) and EDTA were measured at final concentrations of 2 mM, with samples containing no metal ions used as reference controls. The influence of urea (0.1 M, 0.5 M, and 1.0 M), 2-mercaptoethanol (1 mM, 10 mM, and 50 mM), imidazole (10 mM, 50 mM, and 200 mM), and SDS (0.1% *w*/*v*, 0.5% *w*/*v*, and 1.0% *w*/*v*) on the recombinant MgUGE were also determined. The kinetic parameters were determined using Tris-HCl buffer (50 mM, pH 8.0), a reaction temperature of 22 °C, and various concentrations of UDP-galactose (between 10 µM and 2 mM). *K*_m_ and ν_max_ were determined by applying non-linear regression using the Labplot (Version 2.0.1, KDE, Berlin, Germany) data analysis software.

### 4.6. Enzymatic Galactosylation Reaction

Galactosylation reactions were based on the method described by Garcia-Garcia et al. [33], using MgGalT7. A typical reaction was performed on a 100-µL-scale, and consisted of 10 mM *p*NP-α-xyloside, 15 mM UDP-glucose, 1 mM MnCl_2_, 18 µg of MgUGE, and 6 µg of MgGalT7 in Tris-HCl buffer (50 mM, pH 8.0). Reaction mixtures were incubated at 37 °C for 24 h, and the *p*NP-glycosides were then analyzed by HPLC-ESI-MS using the same elution parameters previously applied by Wang et al. for indoxyl-glycosides [34]. In brief, samples were analyzed using reversed-phase HPLC with online UV and ESI-MS detection (Shimadzu MS-2020, Tokyo, Japan). The analytes were separated using a HyperClone 5 µm octadecylsilyl (ODS) column (250 × 4.6 mm, Phenomenex, Tianjin, China), with an ammonium formate eluent (50 mM, pH 4.5, 1 mL/min) mixed with increasing concentrations of acetonitrile (from 10% to 60% in the first 8 min), and then detected at a UV wavelength of 300 nm. The ESI-mass detection was performed using positive ionization, and single ion mode (SIM) at the *m*/*z* values of 294.20 for the detection of *p*NP-xylose, and 456.20 for *p*NP-xylose-β-1,4-galactosyltransferase.

### 4.7. Homology Modeling and Phylogenetic Analysis

A homology model of MgUGE was generated using the structural information of human UGE (Protein Data Bank ID: 1EK6) with the MODELLER homology software (Version 9.17) [35]. The three-dimensional model of MgUGE was generated using Accelrys Discovery Studio Visualizer (Version 4.0, Biovia, Cambridge, UK). The two-dimensional ligand-protein interaction diagrams were created using LigPlot Plus (Version 1.4.) [36]. The positions of the complexed UDP-glucose and NADH from the human UGE crystal data were used to prepare the homology model, and to generated the two-dimensional ligand-protein interaction diagram of the active site of MgUGE. Sequence alignments and phylogenetic analysis based on MUSCLE and PhyML, respectively, were performed using online tools provided by Dereeper et al. [37].

## 5. Conclusions

In this work, an oyster UDP-glucose epimerase was successfully cloned, recombinantly expressed, and biochemically characterized. The temperature and pH optima were in a similar range to those of most reported UGEs. The good thermal stability of the epimerase at temperatures between 16 and 30 °C are an interesting feature for biotechnological applications, and gives MgUGE an advantage over other UGE isoforms in large-scale fermentations of glycosylated products, by saving process energy.

## Supplementary Materials

Supplementary materials can be found at http://www.mdpi.com/1422-0067/19/6/1600/s1.

## Abbreviations

cDNA	Complementary deoxyribonucleic acid
DEPC	Diethylpyrocarbonate
EDTA	Ethylenediaminetetraacetic acid
ESI-MS	Electrospray ionization mass spectrometry
GALK	Galactokinase
GALT	Galactose-1-phosphate uridylyltransferase
HPLC	High-performance liquid chromatography
ID	Identifier
IPTG	Isopropyl-β-D-thiogalactopyranoside
LCMS	Liquid chromatography-mass spectrometry
MgGalT7	β-1,4-Galactosyltransferase derived from *Magallana gigas*
MUSCLE	Multiple sequence comparison by log-expectation
NAD^+^	Oxidized form of nicotinamide adenine dinucleotide
NADH	Reduced form of nicotinamide adenine dinucleotide
PAGE	Polyacrylamide gel electrophoresis
PCR	Polymerase chain reaction
PDB	Protein data bank
PhyML	Phylogenies maximum likelihood
*p*NP	*para*-Nitrophenol
ODS	Octadecylsilyl
RNA	Ribonucleic acid
SDS	Sodium dodecyl sulfate
SIM	Single ion mode
StUGD	UDP-glucose dehydrogenase derived from *Sphaerobacter thermophilus*
UDP	Uridine diphosphate
UGE	UDP-glucose 4-epimerase
