# Supplementary material for: UDP-Glucose 4-Epimerase and β-1,4-Galactosyltransferase from the Oyster Magallana gigas as Valuable Biocatalysts for the Production of Galactosylated Products
Source: Int J Mol Sci. 2018 May 29;19(6):1600. doi: 10.3390/ijms19061600 (PMC6032241; doi:10.3390/ijms19061600)
Supplement: Supplementary file 1 [file ijms-19-01600-s001.pdf]

# Supplementary Materials: UDP-glucose 4-epimerase and beta-1,4-galactosyltransferase from the oyster *Magallana gigas* as valuable biocatalysts for the production of galactosylated products

**Hui-Bo Song, Meng He, Zhi-Peng Cai, Kun Huang, Sabine L. Flitsch, Li Liu and Josef Voglmeir**

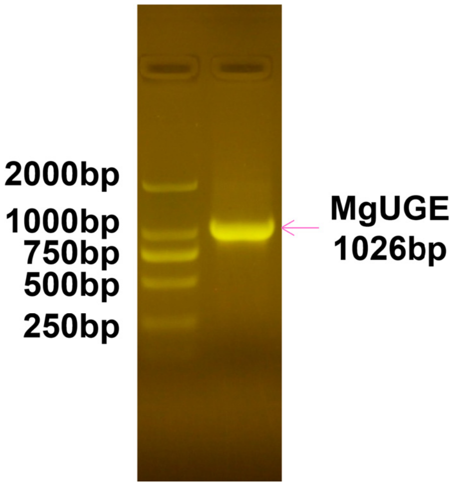


**Supplementary Figure S1:** Agarose gel electrophoresis of the PCR product of MgUGE.

(a)

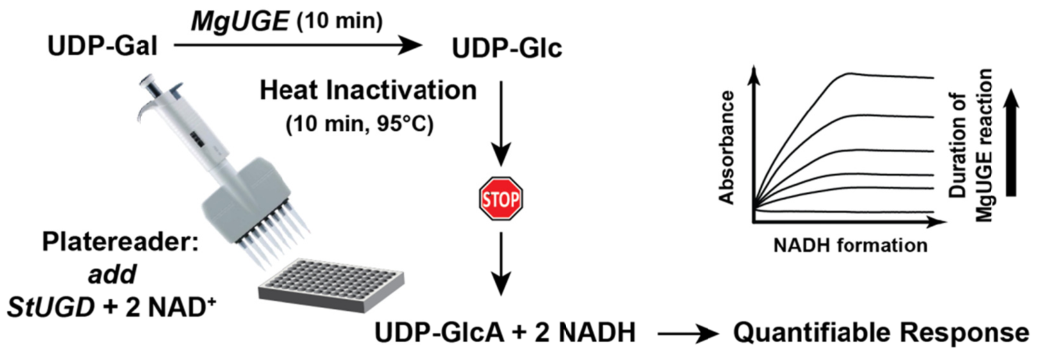


(b)

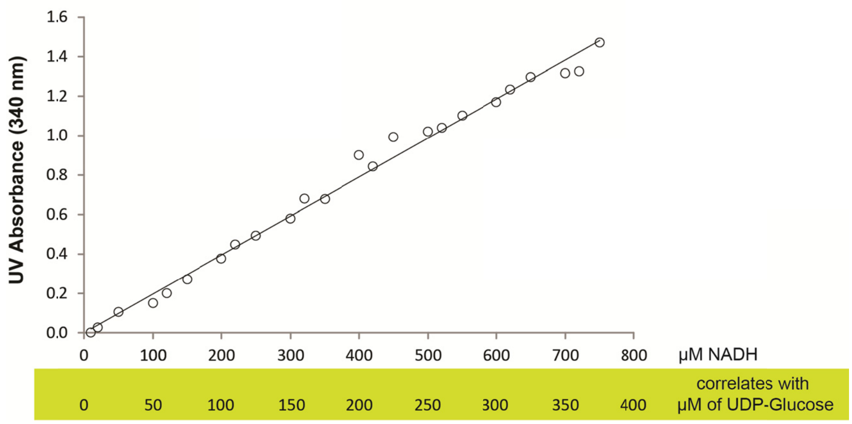


**Supplementary Figure S2.** (a) Schematic overview of the experimental setup for the plate reader-based spectrophotometric detection of MgUGE activity. (b) Relationship between the UV absorbance of various NADH-concentrations, and the concentration of generated UDP-Glucose.

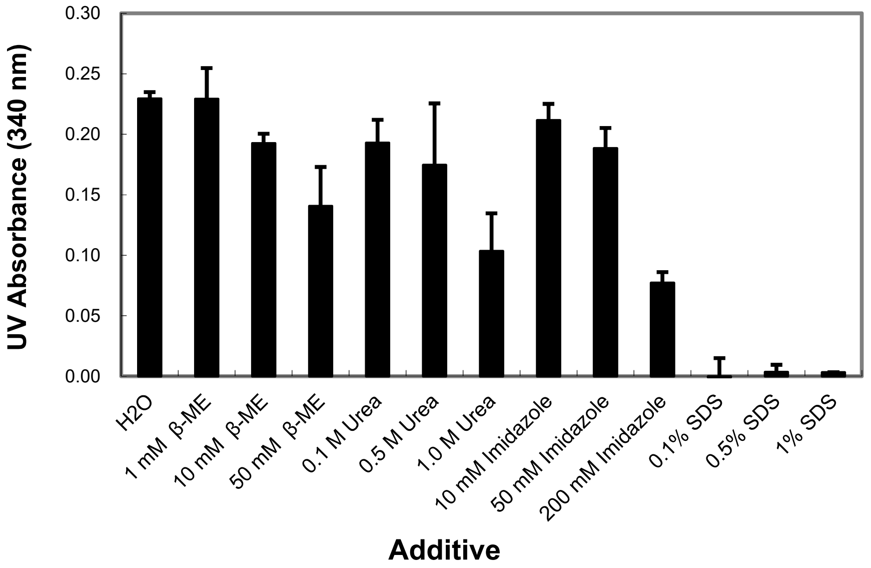


**Supplementary Figure S3:** Impact of additives on the enzymatic activity of MgUGE (β-ME – 2-meracaptoethanol; SDS – sodium dodecyl sulfate).

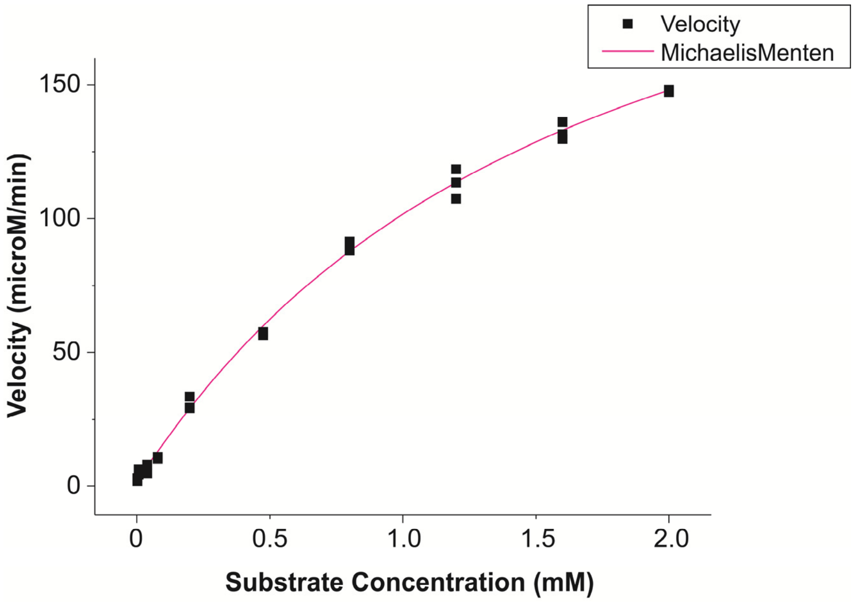


**Supplementary Figure S4:** Michaelis-Menten Plot for the analysis of the kinetic parameters of MgUGE towards the substrate UDP-galactose.

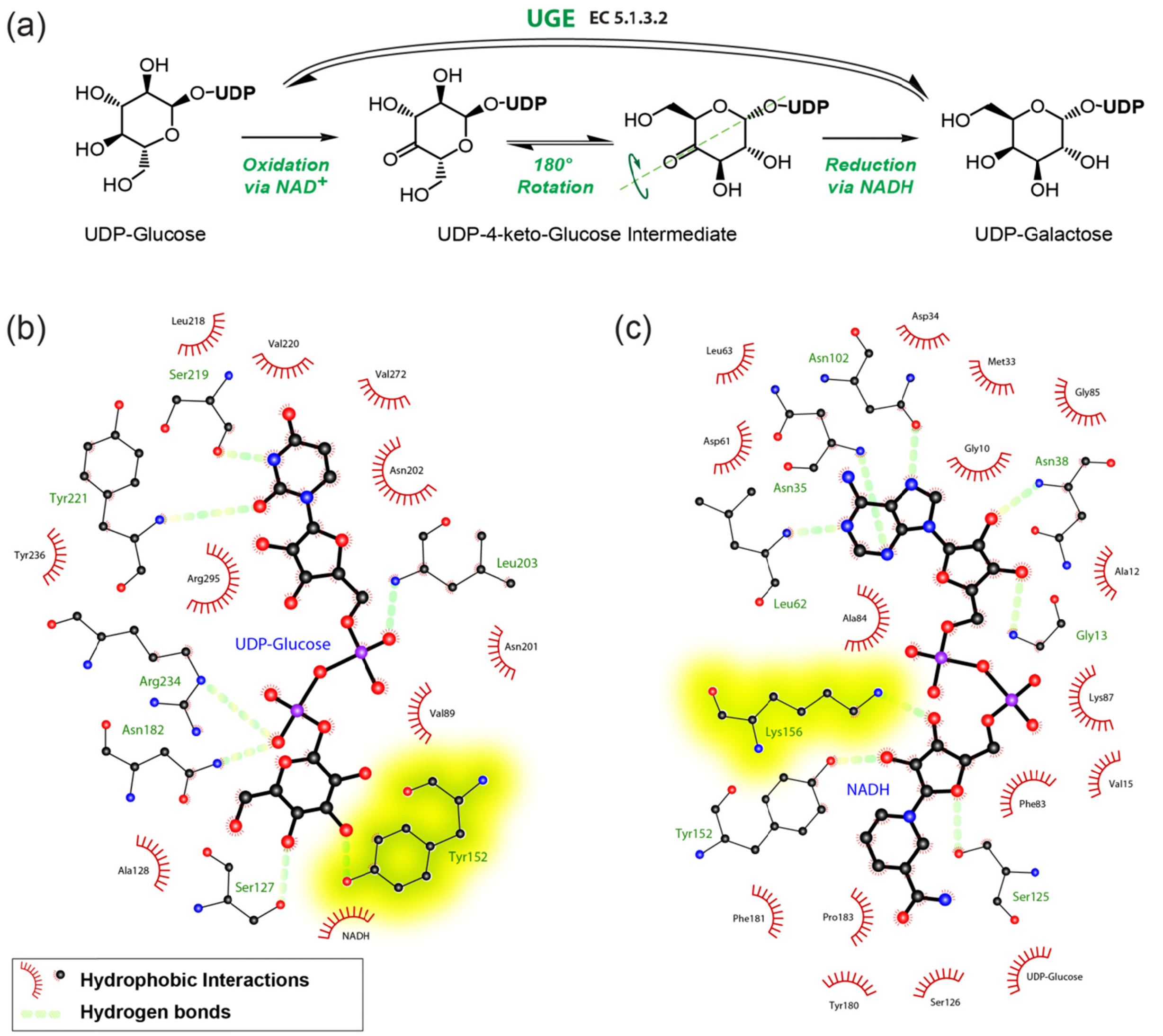


**Supplementary Figure S5. Catalytic mechanism and substrate recognition of UGE. (a)** Reaction mechanism of the substrate interconversion within the active site of UGE; Two-dimensional illustration of the substrate-ligand interactions of MgUGE with **(b)** UDP-Glucose and **(c)** NADH based on crystallographic data of the human UGE isoform. The catalytic amino acids are highlighted in yellow.

```
                 1                                                     59
(K1QYA2)    Mg  MGDSCILVTGGAGFVGSHSVIELIEAGYSVVVMDNLANASM------ESIKRVEEITGKSIPSYS
((Q14376)   Hs  MAEK-VLVTGGAGYIGSHTVLELLEAGYLPVVIDNFHNAFRGGGSLPESLRRVQELTGRSVEFEE
((Q9W0P5)   Dm  MAPPTVLVTGGAGYIGSHTVLEMLNAGYNVICVDNLCNAYSSGAKLPEALSRVQEITGKKVNFYR
((P09147)   Ec     MRVLVTGGSGYIGSHTCVQLLQNGHDVIILDNLCNSKRS-----VLPVIERLGGKHPTFVE-
((C8VAU8)   An  MPSGSVLVTGGTGYIGSFTTLALLEAGYKVVVADNLYNSSA------EALNRIELISGKKAEFAQ

                60                                                            123
(K1QYA2)    Mg  VDLLDKPALSDLFNKHK-FTSVIHFAGLKAVGESCQLPLLYYKNNVGGTVNLLEVMKEKGVKNLI
(Q14376)    Hs  MDILDQGALQRLFKKYS-FMAVIHFAGLKAVGESVQKPLDYYRVNLTGTIQLLEIMKAHGVKNLV
(Q9W0P5)    Dm  VDITDREQVRSVFQEHK-IDMVAHFAALKAVGESCRIPLQYYHNNMTGTNVLLEAMADNNVFKFV
(P09147)    Ec  GDIRNEALMTEILHDHA-IDTVIHFAGLKAVGESVQKPLEYYDNNVNGTLRLISAMRAANVKNFI
(C8VAU8)    An  LDVTDEAAFDKVFEAHPDIDSVIHFAALKAVGESGEKPLDYYHVNVYGTICLLRSMVRHNVTNIV

                124                                                           172
(K1QYA2)    Mg  FSSSATVYGS----PQYLPIDEKHPVGGCTNPYGKTKYFIEEILRDLHKAEPD-----------
(Q14376)    Hs  FSSSATVYGN----PQYLPLDEAHPTGGCTNPYGKSKFFIEEMIRDLCQADKT-----------
(Q9W0P5)    Dm  YSSSATVYGE----PKFLPVTEEHPTGNCTSPYGKTKYFTEEILKDLCKSDKR-----------
(P09147)    Ec  FSSSATVYGD----NPKIPYVESFPTGTPQSPYGKSKLMVEQILTDLQKAQPD-----------
(C8VAU8)    An  FSSSATVYGDATRFPDMIPIPEHCPLG-PTNPYGNTKFAIELAITDVINAQRNNAKKAGNETEAA

                173                                                           236
(K1QYA2)    Mg  -WNIIMLRYFNPVGAHKSGKIGEDPQGIPNNLMPFIAQVAVGKRAELSVYGSDYDTPDGTGVRDY
(Q14376)    Hs  -WNAVLLRYFNPTGAHASGCIGEDPQGIPNNLMPYVSQVAIGRREALNVFGNDYDTEDGTGVRDY
(Q9W0P5)    Dm  -WAVVSLRYFNPVGAHISGRIGEDPNGEPNNLMPYIAQVAVGRRPSLSVYGSDFPTHDGTGVRDY
(P09147)    Ec  -WSIALLRYFNPVGAHPSGDMGEDPQGIPNNLMPYIAQVAVGRRDSLAIFGNDYPTEDGTGVRDY
(C8VAU8)    An  KWNGALLRYFNPAGAHPSGIMGEDPQGVPYNLLPLLAQVATGKREKLLVFGDDYASHDGTAIRDY

                237                                                           299
(K1QYA2)    Mg  IHVVDLAQGHVAAQKKIEK--KCGCKVYNLGTGKGYSVIEMAKAFEEASGKPIPFKKVDRRAGDV
(Q14376)    Hs  IHVVDLAKGHIAALRKLKE--QCGCRIYNLGTGTGYSVLQMVQAMEKASGKKIPYKVVARREGDV
(Q9W0P5)    Dm  IHIVDLAEGHVKALDKLRNIAETGFFAYNLGTGVGYSVLDMVKAFEKASGKKVNYTLVDRRSGDV
(P09147)    Ec  IHVMDLADGHVVAMEKLAN--KPGVHIYNLGAGVGNSVLDVVNAFSKACGKPVNYHFAPRREGDL
(C8VAU8)    An  IHILDLADGHLKALNYLRA-NNPGVRAWNLGTGRGSTVYEMIRAFSKAVGRDLPYEVAPRRAGDV

                300                                                           341
(K1QYA2)    Mg  GSVYGNADLAKEELGWSASRDLKQMCEDTWRWQSSNPNGFRK
(Q14376)    Hs  AACYANPSLAQEELGWTAALGLDRMCEDLWRWQKQNPSGFGTQA
(Q9W0P5)    Dm  ATCYADATLADKKLGWKAERGIDKMCEDTWRWQSQNPNGYANK
(P09147)    Ec  PAYWADASKADRELNWRVTRTLDEMAQDTWHWQSRHPQGYPD
(C8VAU8)    An  LNLTSNPTRANTELGWKAQRTLEQACEDLWLWTKNNPQGYRQQPPAELLEQLKK
```

**Supplementary Figure S6:** Homology alignment of MgUGE with UGE homologues from *Homo sapiens* (Hs), *Drosophila melanogaster* (Dm), *Escherichia coli* (Ec) and *Aspergillus nidulans* (An). The thyrosine and lysine residues highlighted in green are the proposed catalytic amino acids. Uniprot identifiers are shown in parentheses.

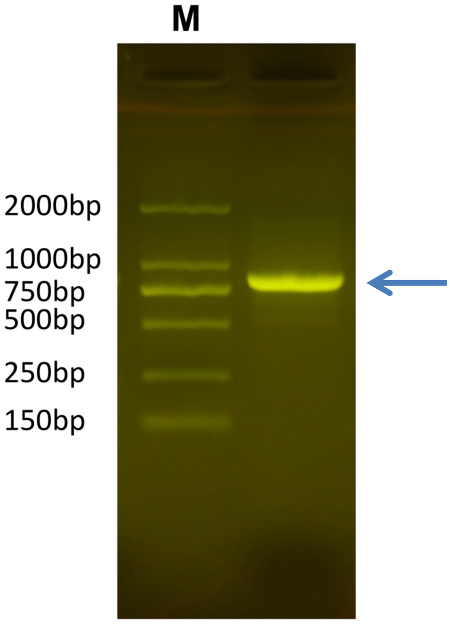


**Supplementary Figure S7:** Agarose gel electrophoresis of the PCR product of MgGalT7.
